# The effect of bitewing radiography on estimates of dental caries experience among children differs according to their disease experience

**DOI:** 10.1186/s12903-018-0596-1

**Published:** 2018-08-09

**Authors:** L. A. Foster Page, D. Boyd, K. Fuge, A. Stevenson, K. Goad, D. Sim, W. M. Thomson

**Affiliations:** 1 Department of Oral Sciences, Faculty of Dentistry, University of Otago, PO Box 56, Dunedin, 9054 New Zealand; 2 Department of Oral Sciences, University of Otago, Dunedin, New Zealand; 3 Hutt Valley District Health Board, Wellington, New Zealand; 4 Waikato District Health Board, Hamilton, New Zealand; 5 Community Oral Health Service, Hutt Valley Hospital, Wellington, New Zealand; 6 Southern District Health Board, Dunedin, New Zealand

**Keywords:** Children, Bitewing radiography, Caries status

## Abstract

**Background:**

Radiography is a regularly used and accepted adjunct to visual examination in the diagnosis of dental caries. It is assumed that not using radiographs can lead to underestimation of dental caries experience with most reports having involved studies of young adults or adolescents, and been focused on the permanent dentition. The aim of this study was to determine the relative contributions of bitewing radiography and clinical examination in the detection of dental caries in primary molars and to determine whether those contributions differ according to caries experience.

**Methods:**

A cross-sectional study was conducted, involving examinations undertaken in dental clinics. Bitewing radiographs taken at the time of the clinical examination were developed and read later, with the data from those used at the analysis stage to adjust the caries diagnosis for the mesial, occlusal and distal surfaces of the primary molar teeth. Children’s clinically determined dmfs score was used to allocate them to one of three caries experience groups (0 dmfs, 1–8 dmfs, or 9+ dmfs).

**Results:**

Of the 501 three-to-eight-year-old children examined, nearly three-quarters were younger than six. Caries prevalence and mean dmfs after clinical examination alone and following radiographs were 63.1% and 4.6 (sd, 6.2), and 74.7% and 5.8 (sd, 6.5) respectively. Among children with a dmfs of 1–8, the number of lesions missed during the clinical examination was greater than the number of 106 (25.6%) in children with a dmfs of 9+. In the 185 children with no apparent caries at clinical examination, 124 lesions were detected radiographically, among 58 (46.8%) of those.

**Conclusions:**

Taking bitewing radiographs in young children is not without challenges or risks, and it must be undertaken with these in mind. Diagnostic yields from bitewing radiographs are greater for children with greater caries experience. The findings of this study further support the need to consider using bitewing radiographs in young children to enhance the management of lesions not detected by a simple visual examination alone.

**Trial registration:**

ACTRN12614000844640.

## Background

Radiography is a regularly used and accepted adjunct in the diagnosis of dental caries. Early studies found using the traditional clinical examination alone that a much lower sensitivity and specificity occurred than when combined with the use of radiographs [1–3]. Not using radiographs has been shown to lead to underestimation of dental caries experience, especially for that in approximal and occlusal surfaces [4–14]. Even in an ideal clinical environment (with good light, and clean and dry teeth), clinical examinations conducted without adjunctive radiography have been shown to underestimate the actual disease level [15]. However, recent studies have questioned whether using radiographs to increase the sensitivity of visual inspection have concomitantly reduced its specificity and introduced a high number of false positives, leading to an overestimation of caries and then overtreatment [16–19].

Most reports on the underestimation of caries without radiographs have involved studies of young adults or adolescents, and have focused on the permanent dentition. In a study of 12-year-old Lithuanian children, the clinical examination alone detected only 60% of approximal cavitated dentine lesions, while, with radiographic examination alone, 90% of lesions were detected. The diagnostic yield improved significantly when the clinical examination was supplemented with radiographs [6]. In young Chinese adults, the use of clinical examination without the use of bitewing radiographs in the posterior teeth led to an underestimation of the number of carious lesions by approximately 50% [11]. Hopcraft and Morgan (2005) investigated the additional diagnostic yield from bitewings for occlusal and approximal dental caries in adults aged 17–30 years. They found the largest underestimation to be for approximal surfaces, where more than two-thirds of approximal dentine caries lesions went undetected by clinical examination. Other studies have focused on populations with high caries experience [2, 13]. In New Zealand adolescents with high caries experience, approximately 40% of carious lesions were missed when radiographs were not used in the caries diagnosis [13].

There have been fewer such investigations in the primary dentition. Newman et al. (2009) investigated the benefits of bitewing radiography in detecting caries in primary teeth in 6–12-year-olds in a non-fluoridated Australian city. They found that visual examination and radiographs detected 48% more proximal primary molar lesions than visual examination alone. In a study of 267 Swedish 5-year-olds with low caries experience (dmfs = 0.4, 85% caries-free) when bitewing radiography was added to the clinical examination, the dmfs rose to 1.2 and only 67% of children were determined to be caries-free. This included adding both enamel and dentine lesions found on radiographs to the clinical examination [8]. Similar findings were reported for Swedish 9-year-olds, with 29% of the children whose primary molars were classified as caries-free using clinical examination found to have caries involving dentine or enamel on bitewing radiographs [10]. In a Dutch study of 50 six-year-old children with high caries experience (mean dmfs of 7.8), clinical examination alone missed approximately 50% of approximal lesions involving dentine [14]. These studies indicate that radiography is also very important for caries diagnosis in primary teeth, but that its yield may differ according to disease level. Many studies that have investigated caries detection methods have not considered the prevalence of the disease in the interpretation of their findings. The increase in sensitivity provided by using radiographs consequently decreases the specificity at times with the number of errors being higher in populations with low caries experience [16].

The American Dental Association and the European Academy of Paediatric Dentistry have produced best-practice guidelines for bitewing radiography in young children [20, 21]. For the primary dentition, it is recommended that individualised radiographic examination be undertaken if proximal surfaces are unable to be scrutinised. Subsequent sets of radiographs should be taken at intervals determined by the child’s caries risk and dentition stage. If the timing of future sets of radiographs is to be determined by the child’s caries status, it is imperative to attempt to take a set of radiographs at an age when the clinician can take advantage of the diagnostic yield that radiographs offer. Although there is evidence to support the use of bitewing radiographs in all children regardless of caries experience, there is debate about the value they provide in the primary dentition, particularly in children who present for clinical examination with an apparently sound dentition. There appears to be concern about the risks of radiation exposure in young children.

The aim of this study was to determine the relative contributions of bitewing radiography and clinical examination in detecting dental caries in primary molars in children in a community with high caries experience, in order to determine whether those contributions differ according to caries experience.

## Methods

A cross-sectional study design was used. Ethical approval for the study was obtained from the Northern B Health and Disability Ethics Committee (14/NTB/39). All three to eight-year-old children attending 37 schools and preschools in New Zealand’s Whanganui region were invited to participate in the study when they attended for their routine clinical examination. Children were excluded if they had recently (within the previous 6 months) had a set of bitewing radiographs taken, had open contacts present between their primary molars, were medically compromised, unable to have radiographs taken, did not have parent consent, or did not assent to participation in the study.

Information on child age, address and ethnicity was obtained from parents. Other socio-demographic information collected included an area-based deprivation score, the NZDep2013. This combines a number of variables measured in the 2013 Census which reflect aspects of social and material deprivation, with each Census meshblock having been allocated a deprivation score [22]. In the current study, the area-based deprivation score was then determined by geocoding each child’s street address and matching it (via meshblock number) to the NZDep2013 data-base. The NZDep2013 allocates each address a deprivation score, ranging from 1 (least deprived) to 10 (most deprived).

Each child underwent a clinical examination in Community Oral Health Service Clinics by one of 13 calibrated dental therapists. Visual examination was conducted under a dental light using a flat dental mirror, explorer and triplex air syringe [23]. Caries status was systematically recorded for each surface. Only the clinical status of the primary dentition was recorded. A carious lesion was counted if cavitation was present. Children’s clinically determined dmfs score was used to allocate them to one of three caries experience groups (0 dmfs, 1–8 dmfs, or 9+ dmfs).

Bitewing radiographs were taken at the time of the clinical examination. Conventional radiography was used, employing the following technical equipment: Belmont Belray 096-C; Girardelli X-30 for the developer; Carestream Insight IP-21 for the film; and Carestream Readymatic for the developer and fixer. Radiographs were developed and read later by two dental specialists (one in paediatric dentistry, the other in dental public health. There is no training of dental radiography specialists in New Zealand, nor anyone registered as a specialist in dental radiography. No magnification was used in reading radiographs, and surfaces that could not be read were recorded as such. The radiographic examiners were calibrated prior to reading radiographs. Radiograph readings for a randomly selected subset of 23 children were used to determine inter-examiner reliability. To determine intra-examiner reliability, each of the two examiners re-read radiographs on a randomly selected subset of 21 children. The intraclass correlation coefficient for dmfs for inter-examiner reliability score was 0.87, and the intra-examiner scores were 0.92 and 0.89, respectively. Children were excluded from the study if the quality of the film was poor or if there was substantial overlap present on the proximal surfaces, making a diagnosis difficult. The radiography data were entered into a separate data-set which was then merged and used at the analysis stage to adjust for the caries diagnosis of the mesial, occlusal and distal surfaces of the primary molars. Only lesions involving dentine were used in the radiographic adjustment.

Data were entered and analysed using the Statistical Package for the Social Sciences (version 23). Following the computation of univariate descriptive statistics, differences among proportions were tested for statistical significance (α = 0.05) using χ^2^ tests; differences with continuous variables were tested for statistical significance using the Wilcoxon signed ranks test.

## Results

Of the 556 children for whom consent was obtained, 55 (9.9%) were excluded following their clinical examination. The final sample comprised 501 three-to-eight-year-old children, of whom approximately one-third were Māori (Table 1). Females comprised almost half of the sample. Nearly three-quarters of the children were under the age of six, and two-fifths resided in a highly-deprived area.

**Table 1:** Sociodemographic characteristics of the sample (parentheses contain row percentages unless otherwise indicated)

	Sex^a^		All^a^
	Female	Male	
Ethnicity			
Māori	78 (32.8)	85 (32.3)	163 (32.5)
NonMāori	160 (67.2)	178 (67.7)	338 (67.5)
Age group (years)			
3–4	35 (14.8)	48 (18.5)	83 (16.7)
5–6	140 (59.3)	146 (56.2)	286 (57.1)
7–8	61 (25.8)	66 (25.4)	127 (25.4)
NZ deprivation score^b^			
1–3 (low)	45 (10.0)	72 (27.4)	117 (23.4)
4–7 (medium)	90 (38.0)	96 (36.5)	186 (37.2)
8–10 (high)	102 (43.0)	95 (36.1)	197 (39.4)
All combined	238 (47.5)	263 (52.5)	501 (100.0)

^a^Column %

^b^This is an area-based SES measure which allocates scores ranging from 1 (lowest deprivation) to 10 (highest deprivation)

Summary estimates of the prevalence and severity of dental caries experience—from the clinical examination only, and then from the clinical examination combined with radiography—are presented in Table 2. The use of radiographs resulted in higher estimates for almost all indicators, except for those involving filled or missing surfaces, and missing teeth. The percent difference between the estimates (computed by dividing the difference between them by the clinical examination value and multiplying the result by 100) ranged from − 12.5 to 90.0%. For the whole-mouth estimates, there were statistically significant differences between the clinical-only and radiographically-adjusted estimates for mean dmft, dt, ft., dmfs, and ds, as well as the whole-mouth caries prevalence estimate, which was higher by 18.2% after radiographic adjustment. For the mesial and distal surfaces only, the prevalence and severity estimates were significantly greater, with almost a one-surface difference (on average) in mean ds being the largest difference observed. With the occlusal surfaces only, there was a significant difference in the prevalence and severity estimates, with a one-third-surface difference in mean ds.

**Table 2:** Comparison of clinical and radiographically adjusted dental caries estimates (brackets contain standard deviation unless otherwise indicated)

	Clinical examination only	Clinical examination and radiographs	Percent difference	*p* value
*Whole-mouth estimates*				
Prevalence^a^	316 (63.1%)	374 (74.6%)	18.2	< 0.001
Severity				
Mean dmft	2.4 (2.8) 0–16	3.3 (3.0) 0–16	37.5	< 0.001
Mean dt	1.6 (2.2) 0–13	2.6 (2.5) 0–13	62.5	< 0.001
Mean mt	0.0 (0.3) 0–5	0.0 (0.3) 0–5	0.0	> 0.009
Mean ft	0.8 (1.4) 0–7	0.7 (1.2) 0–6	−12.5	< 0.001
Mean dmfs	4.6 (6.2) 0–44	5.8 (6.5) 0–45	26.1	< 0.001
Mean ds	2.1 (3.3) 0–26	3.3 (3.8) 0–30	57.1	< 0.001
Mean ms	0.1 (0.3) 0–5	0.1 (0.3) 0–5	0.0	> 0.009
Mean fs	2.5 (5.0) 0–44	2.5 (5.0) 0–44	0.0	> 0.009
*Mesial and distal surfaces of molars only*				
Prevalence^b^	266 (53.1%)	343 (68.45)	28.8	< 0.001
Severity				
Mean dfs	2.0 (2.7) 0–17	2.9 (3.1) 0–17	45.0	< 0.001
Mean ds	1.0 (1.6) 0–11	1.9 (2.1) 0–15	90.0	< 0.001
Mean fs	1.0 (2.1) 0–17	1.0 (2.1) 0–17	0.0	> 0.009
*Occlusal surfaces of molars only*				
Prevalence^b^	250 (49.9%)	283 (56.5%)	13.2	< 0.001
Severity				
Mean dfs	1.6 (2.2) 0–8	1.8 (2.2) 0–8	12.5	< 0.001
Mean ds	0.6 (1.3) 0–8	0.8 (1.4) 0–8	33.3	< 0.001
Mean fs	1.0 (1.7) 0–8	1.0 (1.7) 0–8	0.0	> 0.009

Shown as mean (SD) with range unless otherwise specified

^a^One or more dmft. ^b^One or more dfs

Summary data on the number of lesions detected in the primary molars are presented in Table 3. The total number of lesions detected by clinical examination and radiography was 1245, and the total number of lesions detected by clinical examination alone was 694; 55.7% were detected with both methods. Thus, 44.3% of the lesions present were missed during the clinical examination. Overall, the greatest discrepancy was for approximal surfaces, with clinical examination alone accounting for 49.3% of the number of surfaces detected with clinical examination and radiography (438 out of 889); for occlusal surfaces, clinical examination alone accounted for 74.0% of the number of surfaces detected with clinical examination and radiography (256 out of 346).

**Table 3:** Number of lesions detected with clinical examinations only (a) and clinical and radiographic examination (b), by tooth and surface^1^ in all children

Upper right quadrant							Upper left quadrant					
(b)	7	45	80	123	33	22	16	26	132	72	45	7
(a)	3	28	24	58	24	7	5	18	71	35	32	0
	D	O	M	D	O	M	M	O	D	M	O	D
2nd molar				1st molar			1st molar			2nd molar		
	D	O	M	D	O	M	M	O	D	M	O	D
(a)	2	43	25	84	38	7	4	34	85	24	39	4
(b)	6	56	55	142	41	18	20	41	145	46	58	8
Lower right quadrant							Lower Left quadrant					

*D* Distal, *O* Occlusal, *M* Mesial

^1^Total number detected with radiography alone = 551; total number of lesions detected with clinical examination alone = 694 (55.7% of those detected with both methods)

In order to determine whether the relative contributions of clinical examination and radiography differed according to caries experience, children were allocated to the following ordinal categories of caries experience: 0 dmfs, with 185 children (43.5%); 1–8 dmfs, with 218 children (36.9%); and 9+ dmfs, with 98 children (19.6%). The mean dmfs score for children who were determined at clinical examination to be caries-free (dmfs = 0.0) was actually 5.9 (sd, 6.0) following radiographic adjustment. Determined at clinical examination to be 3.8 (sd, 2.3), the mean dmfs of the medium group (dmfs between 1 and 8) was 6.1 (sd, 7.1) following radiographic adjustment. That for the high–caries-experience group (dmfs 9+) rose from 15.1 (sd, 5.7) to 16.3 (sd, 5.7) following radiographic adjustment.

Data are presented in Table 4 on the number of lesions detected in primary molars in the children in those three different caries experience groups. For those with no apparent caries experience, radiographs detected 124 lesions, of which nearly two-thirds (62.1%) were detected on the distal surface of the first molars. That raised the prevalence of caries in that group from 0.0 to 31.4%. Among children with 1–8 dmfs, the total number of lesions missed during the clinical examination was 214 (38.5%), with the mesial surface of the maxillary right second molar and distal surfaces of the first primary molars showing the greatest discrepancy. The clinical examination alone accounted for only 55.7% of the number of approximal surfaces detected with clinical examination and radiography (234 out of 420), and clinical examination alone accounted for 79.4% of the number of occlusal surfaces detected with clinical examination and radiography (108 out of 136). Among children with a dmfs of 9+, the total number of lesions missed during the clinical examination was 106 (25.6%). Overall, the discrepancy was greatest for approximal surfaces, with clinical examination alone accounting for only 64.8% of the number of surfaces detected with clinical examination and radiography (177 out of 273). For occlusal surfaces, clinical examination alone accounted for 92.9% of the number of surfaces detected with clinical examination and radiography (131 out of 141).

**Table 4:** Number of lesions detected (a) by clinical examination alone and (b) by clinical examination and radiography, by molar tooth and surface, and by caries experience group

Children (*N* = 185) clinically diagnosed with 0 dmfs:												
	*Upper right quadrant*						*Upper left quadrant*					
(b)	0	4	9	20	2	1	0	3	21	7	4	0
(a)	0	0	0	0	0	0	0	0	0	0	0	0
	D	O	M	D	O	M	M	O	D	M	O	D
	Second molar			First molar			First molar			Second molar		
	D	O	M	D	O	M	M	O	D	M	O	D
(a)	0	0	0	0	0	0	0	0	0	0	0	0
(b)	0	1	3	19	2	3	1	1	17	2	3	1
	*Lower right quadrant*						*Lower left quadrant*					
Total number detected with clinical examination and radiography = 124; number detected with clinical examination alone = 0 (0.0% of the latter)												
Children (*N* = 218) clinically diagnosed with 1–8 dmfs:												
	*Upper right quadrant*						*Upper left quadrant*					
(b)	7	45	80	123	33	22	16	26	132	72	46	7
(a)	3	28	24	58	24	7	5	18	71	35	32	0
	D	O	M	D	O	M	M	O	D	M	O	D
	Second molar			First molar			First molar			Second molar		
	D	O	M	D	O	M	M	O	D	M	O	D
(a)	2	43	25	84	38	7	4	34	85	24	39	4
(b)	6	56	55	142	41	18	20	41	145	46	58	8
	*Lower right quadrant*						*Lower left quadrant*					
Total number detected with clinical examination and radiography = 556; number detected with clinical examination alone = 342 (61.5% of the latter)												
Children (*N* = 98) clinically diagnosed with 9+ dmfs:												
	*Upper right quadrant*						*Upper left quadrant*					
(b)	4	17	25	34	18	7	5	13	31	30	14	5
(a)	2	14	12	24	17	4	4	12	24	21	14	0
	D	O	M	D	O	M	M	O	D	M	O	D
	Second molar			First molar			First molar			Second molar		
	D	O	M	D	O	M	M	O	D	M	O	D
(a)	1	18	16	23	22	4	2	17	22	15	17	3
(b)	4	21	31	28	22	4	11	17	31	19	19	4
	*Lower right quadrant*						*Lower left quadrant*					
Total number detected with clinical examination and radiography = 414; number detected with clinical examination alone = 308 (74.4% of the latter)												

## Discussion

This study set out to determine the relative contributions of bitewing radiography and clinical examination in the detection of dental caries lesions in primary molars in children in a community with high caries experience, and to explore this in groups of children with different caries experience. It found that less caries experience was detected when radiographs are not used in caries diagnosis in these young children. When the total number of lesions was taken into account, the actual difference was 44% less disease experience detected. A number of children who were determined to be caries-free at the time of the clinical examination were found not to be so following the reading of radiographs. The extent of the under-estimation of caries experience differed according to how much overall caries experience the child had, with those in the medium caries group (1–8 dmfs) having the greatest underestimation, and those in the highest group having the least.

It is important to consider the study’s strengths and weaknesses. Among the former are the sample’s large size (501 young children) and representativeness, and the comprehensiveness of the data collected. Caries data were collected at surface level rather than tooth level, and the clinical examinations were undertaken in appropriate conditions by trained calibrated clinicians. Radiographs were read by two calibrated dental specialists to ensure accuracy, with inter- and intra-examiner reliability being excellent. Another strength of this study is that it takes a pragmatic approach to the research question in reporting data from a number of clinicians working in a community setting rather than data from a small number of trained examiners in an ideal clinical environment, as used in other studies [17]. A weakness was the lack of data on examiner reliability for the dental therapists conducting the clinical examinations. The burden on the young children to have to participate in another examination (for examiner calibration) was felt to be unfair due to their very young age and lower ability to cooperate.

This is the first study to report on the contribution of bitewing radiography to caries diagnosis in children as young as three and on whether those contributions differ according to caries experience on presentation at clinical examination. A hierarchical model of efficacy exists for appraising the literature on the efficacy of imaging. This study reports at Level 2. This addresses diagnostic accuracy, sensitivity, and specificity associated with interpretation of the images, and it includes the person(s) interpreting the image as well as the images per se [24]. Some 369 of the children were six years old or younger and able to have a set of quality radiographs taken. Of the 55 children excluded, only 20 were unable to have radiographs taken or the films could not be read (4% of the overall sample of 521). This differs from a previous study, where it was found impossible to take bitewing radiographs for 18% of 5-to-6-year-olds, with a further 14% of the surfaces being unreadable on those which were taken [14]. By contrast, all clinicians in the current study had training in beam angulation when using adhesive soft foam bitewing tabs; these have been shown to be well-tolerated by young children [17], and may have been responsible for both the high number of bitewing films being taken and their excellent quality.

The findings indicate that using a simple visual clinical examination alone in young children is likely to lead to substantial underestimation of caries experience, and that this effect differs with the extent of the clinically-apparent caries experience. At whole-mouth level, radiographic diagnosis made a moderate difference to the estimates of caries experience, but the surface-specific caries experience data indicate that radiographic diagnosis made a considerable difference, particularly for approximal surfaces. When the total number of lesions involving dentine was taken into account, clinical examination alone resulted in underestimation by 44%. This differential is similar to that observed with a group of 242 Australian 6-year-olds, where a 48% underestimation was found with lesions that involved the inner half of enamel and dentine [2].

Previous studies have suggested that bitewing radiography has the greatest value in children with the highest susceptibility to caries [2]. However, findings with apparently low-caries-risk children have shown that, even if they were diagnosed as caries-free following a clinical examination, nearly one-quarter were found to present with a caries lesion following a bitewing radiograph [8, 10]. Other studies in low-caries-risk populations suggest that taking radiographs does not improve the ability to detect caries lesions when operative treatment is to be undertaken, and that the caries experience of the wider community should be considered in developing locally appropriate guidelines on the use of radiographs [17, 20, 21]. In the current study, almost one-third of those who were apparently “caries-free” were found on radiographic examination to actually have dental caries lesions. This finding is similar to those from a smaller study from the Netherlands where, of the 13 children residing in a high-caries-experience community who were found to be caries-free after a clinical examination, 5 (38%) actually had one or more caries lesions involving dentine [14]. Our study did not include caries lesions limited to the enamel, so the findings may have substantially underestimated the true disease level.

For children with greater caries experience, the differences observed were even more marked, with approximately one-quarter of lesions missed in children presenting with high caries experience. In terms of differences by tooth surface type, the distal surface of the first primary molar consistently yielded the greatest underestimation of dental caries, with 45% of lesions at that site missed. That children who presented with the greatest disease levels had the lowest number of extra lesions found with radiographs is not surprising, given that most will have presented with primary molars that are cavitated or already restored, both of which are more easily identified during the clinical examination. The majority of lesions were found on approximal surfaces, supporting previous studies showing these to be the most commonly missed [8–13, 25]. Identifying such lesions early in young children is important because it allows non-operative preventive measures to be undertaken, and also so that children can be more accurately assigned a caries risk status. This suggests that a simple clinical examination alone does not give a true representation of a young child’s caries status, especially for approximal surfaces. A thorough visual and tactile examination using criteria that take the dynamic nature of the disease into consideration and evaluate its activity needs to be considered, because this has been shown to be the method of choice in daily practice for detecting caries in primary teeth [18].

In considering the relevance of these findings in relation to the sensitivity and specificity of diagnostic tests, it has to be remembered that caries diagnosis at the person level is not an “all or nothing” event. That is, it may be so at the individual surface level, but the summary measure (dmfs) is aggregated up at the person level as a continuum (albeit one based on a count variable). Thus, we have a binary event (sound versus decayed) at the surface level, but a continuous variable at the person level. What does this mean? Consider the first group of children in Table 4: those 185 individuals were deemed to be caries-free in the clinical examination, yet 58 (31.4%) actually had one or more carious lesions. Calculating the sensitivity and specificity of the clinical examinations alone (and treating the clinical examination plus radiography as the true situation) at the person level gives 100% specificity and 0% sensitivity, which is not very useful. We cannot do a similar calculation for the other two groups because the person-level prevalence of the condition is 100% in each of those groups anyway. Another key issue is the relative importance of a false positive and a false negative: for a given surface, a false positive may result in a surgical intervention through the placement of the restoration that was not actually needed; a false negative may result in disease progression and sequelae such as pain, infection, the collapse of the tooth structure, the loss of the tooth through extraction, and so on.

Further research that involves clinical trials (not just cross-sectional studies) to explore how radiographic examination impacts on diagnosis (and the treatment decisions) for caries lesions in primary molars of children with different caries experience would be of benefit [26], as would meta-analysis and synthesis of the findings of studies such as the current one.

Finally, it should be borne in mind that the focus of this study was the lesions themselves rather than their prevention or restorative treatment. We are not necessarily advocating restoration of all of the extra lesions discovered through using radiography. Whether that takes place remains a clinical decision to be made by the treating clinician. After all, it is not possible to determine from a radiograph whether a lesion has cavitated (unless it is very advanced), and so it would be incumbent upon the treating clinician to confirm the extent of the lesion before deciding on the most appropriate way to manage it. Nevertheless, it is likely that a considerably higher proportion of such lesions in the primary dentition will have cavitated. The main focus of our paper is on the diagnostic yield, irrespective of the action implications of that yield.

## Conclusion

Taking bitewing radiographs in young children is not without challenges or risks, and it must be undertaken with these in mind. Diagnostic yields from bitewing radiographs are greater for children with greater caries experience. The findings of this study further support that using bitewing radiographs in young children detect more lesions than a simple visual examination alone. Children residing in a community with high caries experience, who present with no clinically detected caries lesions following a simple visual examination and who are unable to tolerate bitewing radiographs being taken, may best be considered at risk of caries when determining the timing of recall. They should be managed preventively until a set of radiographs can confirm that they have no caries lesions present.

## Abbreviations

dmfs: decayed missing and filled surfaces in the primary dentition
NTB: Northern B Health and Disability Ethics Committee
NZDep2013: New Zealand deprivation scores based on 2013 Census data
χ2 tests: chi squared tests
